# Evaluation between the Usability and Physicochemical Property of Acyclovir Ointments

**DOI:** 10.1155/2018/8761394

**Published:** 2018-08-01

**Authors:** Yutaka Inoue, Akiho Mitsumori, Itsuka Shinohara, Sachie Narumi, Isamu Murata, Ikuo Kanamoto

**Affiliations:** Laboratory of Drug Safety Management, Faculty of Pharmacy and Pharmaceutical Sciences, Josai University, 1-1 Keyakidai, Sakado-shi, Saitama 3500295, Japan

## Abstract

A lot of prescription medicines have become switch over-the-counter (OTC) medicines. However, additives in brand-name drugs, generic drugs, and switch OTC drugs differ; therefore, the feelings associated with the use of these medicines vary for patients. The aim of this study was to compare the physicochemical properties and the feeling of use (assuming skin as an index of usability) of acyclovir (ACV) ointments. Five ACV ointments were used: ACV-A, a brand-name drug, ACV-B and ACV-C, generic drugs, and ACV-D and ACV-E, switch OTC drugs. The physicochemical properties were evaluated by determining the content uniformity, water content, flattening, viscosity and viscoelasticity, and near-infrared (NIR) absorption spectroscopy. Skin friction was measured to evaluate the feeling associated with use. Results of the content uniformity test indicated that the ACV content was uniform, and equivalence was observed. Measurement of moisture content indicated that this parameter differed in each ointment preparation. The yield value, which was calculated by measuring flattening, was 4416.7 dyne/cm^2^ for ACV-A, 1175.7 dyne/cm^2^ for ACV-B, 2114.9 dyne/cm^2^ for ACV-C, 4234.5 dyne/cm^2^ for ACV-D, and 3620.7 dyne/cm^2^ for ACV-E. Measurement of viscosity and viscoelasticity revealed that viscosity increased with time and the viscoelasticity of each ointment. The second derivative of the NIR spectrum revealed that ACV-B and ACV-C had a wider spectrum of absorption than the other ointments. ACV-B had lesser friction than other ointments. These findings suggest that differences in the type and content of additives (macrogol) result in differences in the physicochemical properties of individual ointments.

## 1. Introduction

A lot of prescription medicines become switch OTC drugs, making it possible to purchase these medicines without prescription with instructions from a pharmacist for self-medication [1, 2]. Famotidine, a gastric acid secretion inhibitor, and indomethacin, an anti-inflammatory drug, are switch OTC drugs for internal medicine, and terbinafine hydrochloride, an antifungal drug, and vidarabine, an antiviral drug, are switch OTC drugs for external medicine. These medicines require pharmacotherapy for patient care through a counter service or a recommendation from a pharmacist to receive a medical examination [3]. Generic drugs can also be prescription drugs and are prescribed as needed by the patient [4]. However, the additives contained in brand-name drugs, generic drugs, and switch OTC drugs differ; therefore, patient experience of these drugs varies.

For example, physicochemical properties of vidarabine were evaluated using brand-name drugs, generic drugs, and switch OTC drugs, and differences in the viscosity of each formulation due to differences in additives in the formulation were reported [5]. Thus, the additives in brand-name drugs, generic drugs, and switch OTC drugs differ and the feelings associated with the use of different drug formulations must be considered. Thus, pharmacists can advise on self-medication by providing instructions to patients through an understanding of the physicochemical properties of each formulation. However, currently, a few reports have compared the physicochemical characteristics of brand-name drugs, generic drugs, and switch OTC drugs.

Acyclovir (ACV) is a commonly used guanine analog antiviral drug. It assumes its active form when phosphorylated in infected cells and subsequently prevents viral growth [6, 7]. ACV is used for the prevention and treatment of herpes simplex virus I (cold sores) and herpes simplex virus II (genital herpes) infections. ACV is used as an internal medicine, ointment, and cream. Also, these ACV formulations are available as both brand-name and generic drugs. Differences in the viscosity and viscoelasticity of brand-name and generic ACV creams have been previously reported [8]. Recently, switch OTC drugs for ACV ointments, which require pharmacist guidance, have been used.

Previously, these ACV creams have reported on the viscosity and incompatibility of original drugs and generic products [8]. However, in this study, ACV ointment was used to evaluate physical properties of OTC drugs as well as prescription drugs (original drugs and generic products). In order to predict an indication of usability in clinical, performing the evaluation due to friction meter is believed to play a role in providing information to healthcare professionals and patients. In this study, the physicochemical properties of ACV formulations were examined: Zovirax® Ointment 5%, a brand-name drug, Acyclovir Ointment 5% “TEVA,” Aciclovir Ointment 5% “TOWA,” a generic drug, and Activir® and Hifuru AC, switch OTC drugs. The major additive contained in the brand-name, generic, and switch OTC ACV ointments is macrogol. It has been reported that the viscosity of polyethylene glycol, a macrogol, varies depending on the average molecular weight and that differences in the feelings associated with the use of the preparation vary when polyethylene glycol is used as an additive [9]. Therefore, differences in the average molecular weight of macrogol were assessed, and the viscosity and viscoelasticity of each ointment were evaluated. Additionally, the skin friction of each ointment was measured, and the feeling of use on the skin was evaluated. Furthermore, to investigate the molecular behavior of the additive in each formulation, the near-infrared (NIR) absorption spectrum was measured. It would be considered to become an item in visualization information of each ointment difference, so mapping by main analysis statistical analysis was evaluated.

## 2. Materials and Methods

### 2.1. Materials

Commercial formulations of ACV ointments were purchased. The brand-name ointment was Zovirax® Ointment 5% (GlaxoSmithKline Co., Ltd.); generic ointments were Acyclovir Ointment 5% “TEVA” (Teva Takeda Pharma Ltd.) and Acyclovir Ointment 5% “TOWA” (Towa Pharmaceutical Co., Ltd.); and OTC ointments were Activir® (GlaxoSmithKline Consumer Healthcare Japan Co., Ltd.) and Hifuru AC (Bankyo Pharmaceutical Co., Ltd.). The properties and additives of each formulation are shown in Table 1. ACV crystal was purchased from Tokyo Kasei Co., Ltd. Other reagents were of special commercial grade (Wako Pure Chemical Industries Co., Ltd.).

### 2.2. Methods

#### 2.2.1. Content Uniformity Test

For the assay, approximately 100 mg of each ointment was weighed; 12.5 mL of diluted sodium hydroxide solution and 37.5 mL of distilled water were added and dissolved. Then, 0.1 mol/L hydrochloric acid solution was added to 7.5 mL of each ointment solution to make 100 mL and test solutions were prepared. Approximately 10 mg of ACV was weighed; diluted sodium hydroxide solution was then added to reach 10 mL. Then, 7.5 mL of diluted sodium hydroxide solution was added to the ACV solution, and distilled water was added to make 50 mL. Hydrochloric acid solution (0.1 mol/L) was added to reach 100 mL and a standard solution was prepared. Measurements were made using a UV-vis recording spectrophotometer (Shimadzu: UV-2500PC). Absorbance of test and standard solutions was measured at a wavelength of 255 nm using 0.1 mol/L hydrochloric acid as a control.

The amount of each ACV ointment was calculated using the following equation:

(1) $$\begin{array}{c} Amount\;\;of\;\;ACV\left(\;mg\;\right)=WS*\frac{AT}{AS}*\frac{1}{2} \end{array}$$

where WS is weighed amount of ACV standard product converted into dehydrate (mg), AT is absorbance of test solution, and AS is absorbance of standard solution.

#### 2.2.2. Measurement of Water Content

Water content was measured using a Karl-Fisher moisture content meter (Kyoto Electronics Manufacturing Co., Ltd.: MKV-710M). KEM AQUA TR-3 (Kyoto Electronics Manufacturing Co., Ltd.) was used as the titrant and KEM AQUA FAT (Kyoto Electronics Manufacturing Co., Ltd.) served as the dehydrated solvent. The water content in 0.03 g of each sample was measured three times at 25°C.

#### 2.2.3. Measurement of Flattening

Spreadability was measured using a spread meter (Rigo) with a measuring temperature of 25°C. Spread diameter was measured after 5, 10, 60, 120, 180, 240, 300, 360, 600, and 900 s.

The yield value was calculated from the following formula using the spread diameter after 180 s:

(2) $$\begin{array}{c} F=47040*G*\frac{V}{\pi^{2}}*D^{5} \end{array}$$

where F is yield value (dyne/cm^2^), G is glass plate weight (115.5g), V is sample size (cm^3^), and D is diameter (mm) when sample spreading stopped.

#### 2.2.4. Viscosity and Viscoelasticity

Dynamic viscosity was measured using a type-E rotational viscometer (Toki Sangyo: TVE-20H). The dynamic viscosity of 0.02 mL of each ointment was measured for 553 s at 25°C using the viscometer with a 3° × R9.7 cone rotor. Measurements were made at a shear rate of 0.01-0.08 → 0.08 -0.01 s^−1^ (0.01 s^−1^: 60 s, the other shear rate: 30 s). The viscosity was measured at a shear rate of 0.08 s^−1^ for 276 s. Viscoelasticity measured the recovery and stress at a shear rate of 1 s before the shear rate changes. The viscoelasticity of each ointment was measured three times under the same conditions.

#### 2.2.5. Near-Infrared Absorption Spectroscopy

NIR absorption spectra were recorded using a Fourier-transform near-infrared analyzer (Buchi NIRFlex N-500). Spectroscopy was performed at a wavelength of 1000-2500 nm and a wavenumber of 10,000-4000 cm^−1^, and spectra were recorded for 8 s at 25°C. Each ointment was placed in a sample cup, and the NIR absorption spectra were measured at 1 nm intervals.

#### 2.2.6. Skin Friction Measurement

The skin friction produced by each ointment was measured using the Frictiometer (Courage + Khazaka Electronic GmbH: FR 700). A 0.1 g sample of each ointment was applied to artificial skin, and skin friction at 25°C was measured by agitation for 20 s at 25 rpm.

#### 2.2.7. Statistical Analysis

Statistical analysis was performed using Tukey's test, with p<0.05 considered to indicate a significant difference.

## 3. Results and Discussion

### 3.1. Content Uniformity Test

A uniformity of content test was conducted to assess the equivalence of ACV content in each ointment (Table 2). The percentage ACV in ACV-A, ACV-B, ACV-C, ACV-D, and ACV-E was 102.1 ± 2.0, 100.8 ± 1.8, 100.0 ± 1.4, 103.1 ± 0.310, and 101.8 ± 0.8%. Tukey's test revealed no significant difference in the uniformity of content in the five ointments. Thus, the contents of ACV in ACV-A, ACV-B, ACV-C, ACV-D, and ACV-E were considered uniform.

### 3.2. Measurement of Moisture Content

The moisture content of each formulation was determined. The results indicated that ACV-A had a moisture content of 1.36 ± 0.27%, ACV-B had a moisture content of 3.22 ± 0.75%, ACV-C had a moisture content of 3.52 ± 0.43%, ACV-D had a moisture content of 1.29 ± 0.19%, and ACV-E had a moisture content of 4.56 ± 0.94%. This indicated that the moisture content in each formulation differed.

### 3.3. Measurement of Flattening

**Figure 1:**
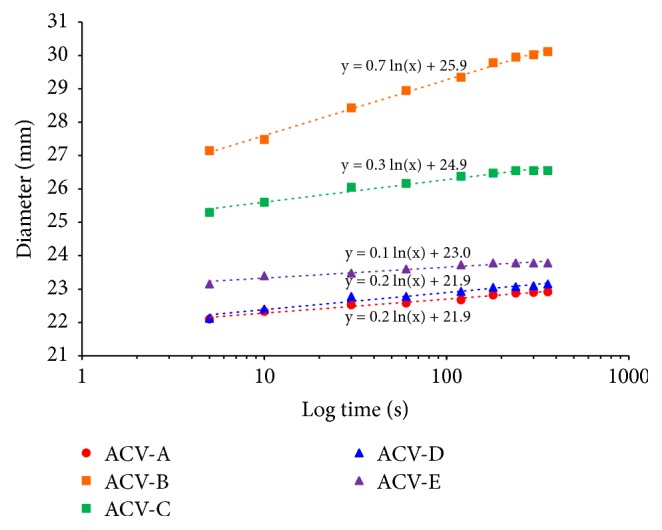
Average diameter of ACV ointments in Logarithm time.

**Figure 2:**
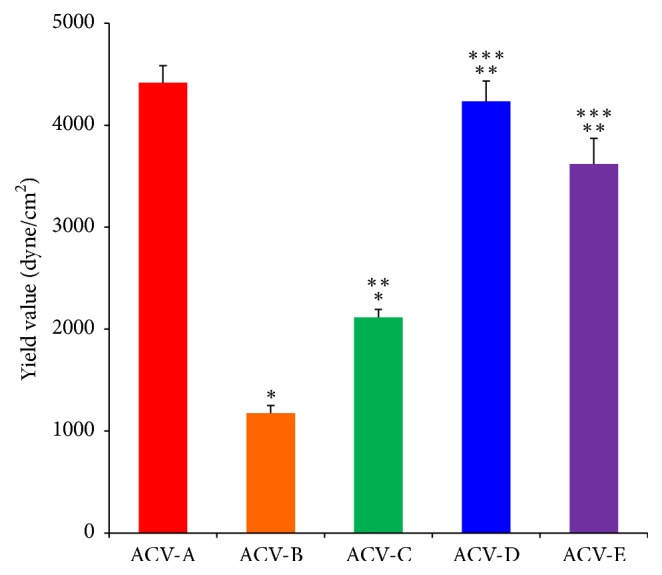
Yield value of ACV ointments at 25°C. Value of the spreadability after 180 seconds (mean ± SD, n=3). Tukey's test was given for official approval (p<0.05). *∗* indicates that significant differences were seen between ACV-A and ACV-B and between ACV-A and ACV-C (p<0.05 for each). *∗∗* indicates that significant differences were seen between ACV-B and ACV-C, between ACV-B and ACV-D, and between ACV-B and ACV-E (p<0.05 for each). *∗∗∗* indicates that significant differences were seen between ACV-C and ACV-D and between ACV-C and ACV-E (p<0.05 for each).

The feeling associated with the use of a semisolid preparation can be evaluated by assessing extensibility and hardness, which are indexes of spreadability and yield value, using a spread meter [10]. Therefore, the spreadability and yield value of each ointment preparation were calculated using a spread meter, and the physical properties were evaluated (Table 3 and Figures 1 and 2). The spreadability was evaluated by comparing the diameters at 180 s when the spreading of each ointment preparation was stable. The diameter of the ointment spread in 180 s was 22.9 mm for ACV-A, 29.8 mm for ACV-B, 26.5 mm for ACV-C, 23.1 mm for ACV-D, and 23.8 mm for ACV-E. From this, the spread of ACV-B was found to cover a wider diameter compared to that of the other ointments. A similar spread of about 23 mm in diameter was found for ACV-A, ACV-D, and ACV-E. Next, the yield values of ACV-A, ACV-B, ACV-C, ACV-D, and ACV-E, which are indexes of hardness, were calculated. The yield value was 4416.7 dyne/cm^2^ for ACV-A, 1175.7 dyne/cm^2^ for ACV-B, 2114.9 dyne/cm^2^ for ACV-C, 4234.5 dyne/cm^2^ for ACV-D, and 3620.7 dyne/cm^2^ for ACV-E. The spreadability and yield value indicated that ACV ointments are soft and extensible preparations in the order of ACV-B > ACV-C > ACV-E > ACV-D > ACV-A. In addition, the spread diameters of ACV-A and ACV-D were similar, and ACV-A and ACV-D exhibited hardness and extensibility compared with other ointments. The extensibility of ointment is due to the additives contained in the preparation [11]. Thus, the differences in spreadability and yield value of each ointment affected the molecular weight and macrogol content, which is an additive contained in each ointment.

### 3.4. Measurement of Viscosity and Viscoelasticity

**Figure 3:**
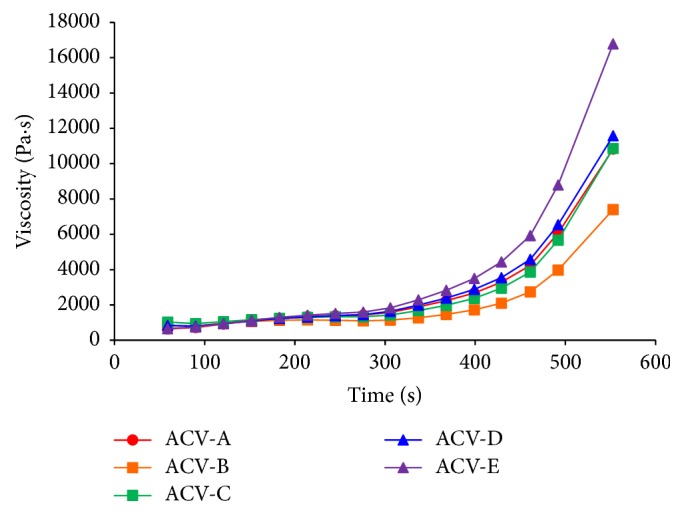
Viscosity curves of ACV ointments at 25°C.

Viscosity was measured to evaluate the viscosity characteristics of each ointment (Figure 3). After 276 s, ACV-A had a viscosity of 1428.8 Pa·s, ACV-B had a viscosity of 1092.8 Pa·s, ACV-C had a viscosity of 1323.1 Pa·s, ACV-D had a viscosity of 1450.0 Pa·s, and ACV-E had a viscosity of 1588.6 Pa·s. There was a small increase in the viscosity of ACV-B over time compared with the other ointments. Thus, the ACV-B formulation undergoes little change in internal structure when subjected to shear stress. Conversely, the viscosity of ACV-E changed markedly over time compared with that of the other ointments. In addition, the viscosity of ACV-A, ACV-C, and ACV-D similarly increased with time. The differing changes in viscosity over time between ointments did not confirm the results obtained for moisture content and spreadability. As such, the varying changes in viscosity with time may have been due to differences in the molecular weight and content of the additive macrogol.

**Figure 4:**
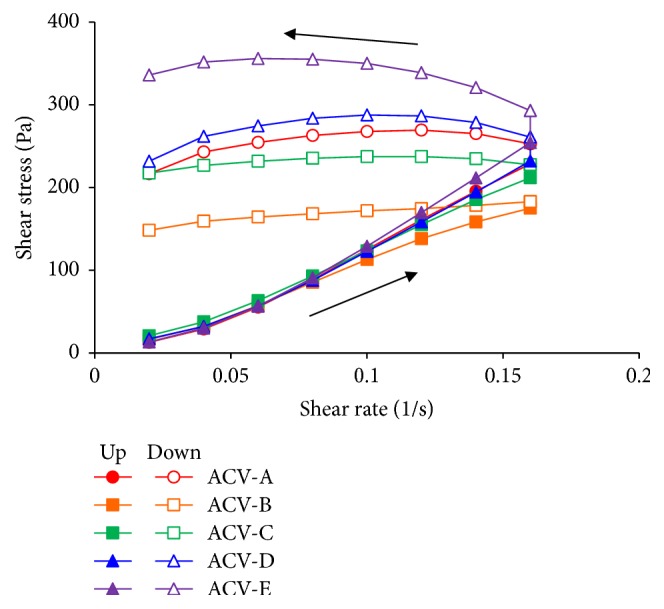
Shear stress versus shear speed curves of ACV ointments.

The viscosity of each ointment preparation differed; therefore, the viscoelasticity of each ointment preparation was evaluated (Figure 4). The shear stress of ACV-B was low, while that of ACV-E was high compared with the other ointments. In addition, the shear stresses of ACV-A, ACV-C, and ACV-D were similar. These results indicate that ACV-B has properties of softness compared with the other formulations. Conversely, ACV-E has properties of strength compared with the other formulations. This was correlated with viscosity.

The rheogram observed with an increase in shear rate differed from that observed with a decrease in shear rate, and a hysteresis loop was observed for all ointment formulations. The presence of a hysteresis loop indicates that time is needed to recover the internal structure, which changed when the shear rate increased [12]. In addition, it is possible to evaluate the thixotropy, that is, the strength of the internal structure by the difference in the area of the hysteresis loop of flow curves when the shear rate is up and down. The area of the hysteresis loop of ACV-B was small, and the area of the hysteresis loop of ACV-E was large compared with that of the other ointment preparations. From the results of the viscosity measurement, the stickiness was confirmed by adding the shear stress. Thus, from the results of viscosity and viscoelasticity, ACV-B was inferred to undergo less change in its internal structure when subjected shear stress and to have properties of softness compared with the other ointments. Conversely, the internal structure of ACV-E was easily changed by shear stress, with a harder formulation compared with the other ointments.

**Figure 5:**
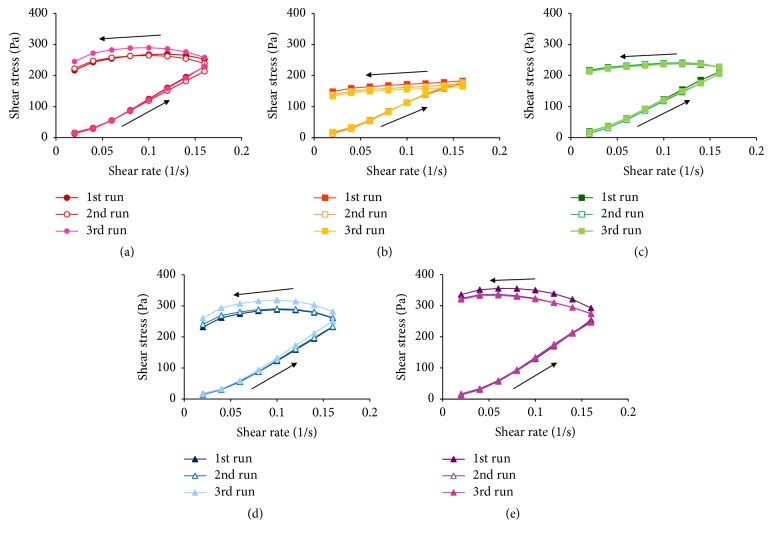
Viscoelasticity curves of each ACV ointment at repeated measurements. (a) ACV-A, (b) ACV-B, (c) ACV-C, (d) ACV-D, and (e) ACV-E.

Next, repeated measurements were performed to evaluate changes in the robustness, viscosity, and viscoelasticity of each ointment (Figure 5). When ACV-E was subjected to repeated shear stress, the shear stress decreased in the third run compared with the first and second runs. Thus, the internal structure of ACV-E may be susceptible to disruption when ACV-E is subjected to repeated shear stress. In addition, the shear stress of ACV-B and ACV-C underwent minimal change even under repeated shear stress. Thus, ACV-B and ACV-C were considered to possess viscoelasticity, and the internal structure of ACV-B and ACV-C could be maintained, even under shear stress. Additionally, the shear stress of ACV-A and ACV-D increased in the third run compared to that in the first and second runs. Thus, when ACV-A and ACV-D were subjected to repeated shear stress, there was less disruption to their internal structures.

The results obtained for viscosity and viscoelasticity differed from those obtained for spreadability and yield value. This suggests that there are differences in the extensibility and change in the internal structure of each ointment. The viscosity and viscoelasticity of cream preparations have been reported to vary depending on the proportion of oil contained in cream [13]. Therefore, in this study, differences in viscosity and viscoelasticity among the ointment preparations were due to differences in moisture content and the molecular weight and content of macrogol. In addition, from the results obtained thus far, ACV-A and ACV-D appear to have similar properties. Thus, macrogol, which is contained by ACV-A and ACV-D as an additive, accounted for their similar molecular weight and content.

### 3.5. Near-Infrared Absorption Spectroscopy

**Figure 6:**
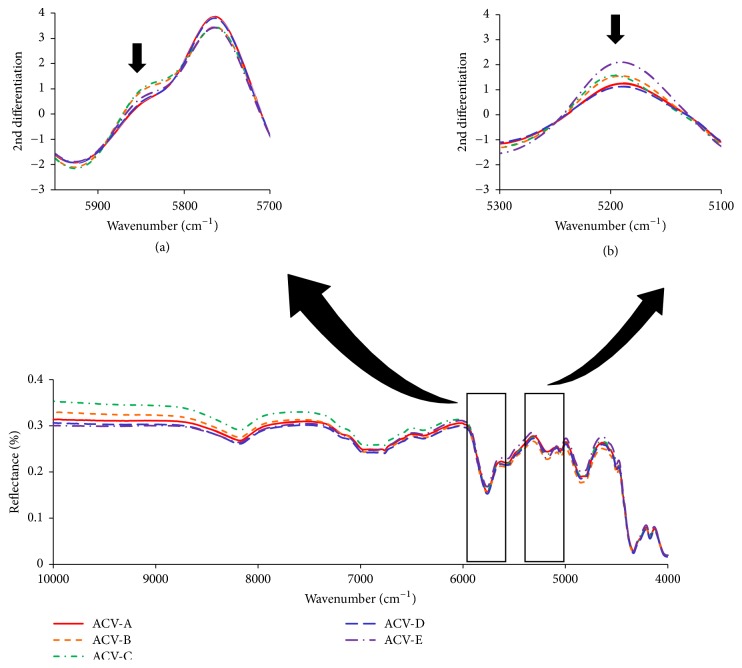
Near-infrared absorption spectra of ACV ointments, observed to 4000-10000 cm^−1^. (a) 2nd differential near-infrared absorption spectra of ACV ointments, observed to 5700-5950 cm^−1^. (b) 2nd differential near-infrared absorption spectra of ACV ointments, observed to 5100-5300 cm^−1^.

NIR absorption spectra were recorded to verify differences in the water or oil content of each formulation (Figure 6). The absorption spectrum of the olefin group (-CH_2_) in the oleaginous base was observed at 4300 and 5800 cm^−1^, and that of the hydroxyl group (-OH) caused by moisture was observed around 5200 cm^−1^. The current study evaluated the oil and water content of each formulation focusing on these spectra [14, 15]. The second derivative of the NIR absorption spectrum revealed that ACV-B and ACV-C possessed a wider absorption spectrum than the other ointments due to the olefin group (Figure 6(a)). Moreover, ACV-E possessed a larger spectrum than the other ointments due to the hydroxyl group (Figure 6(b)). From these results, differences in oleaginous base and moisture content of each formulation were clarified.

**Figure 7:**
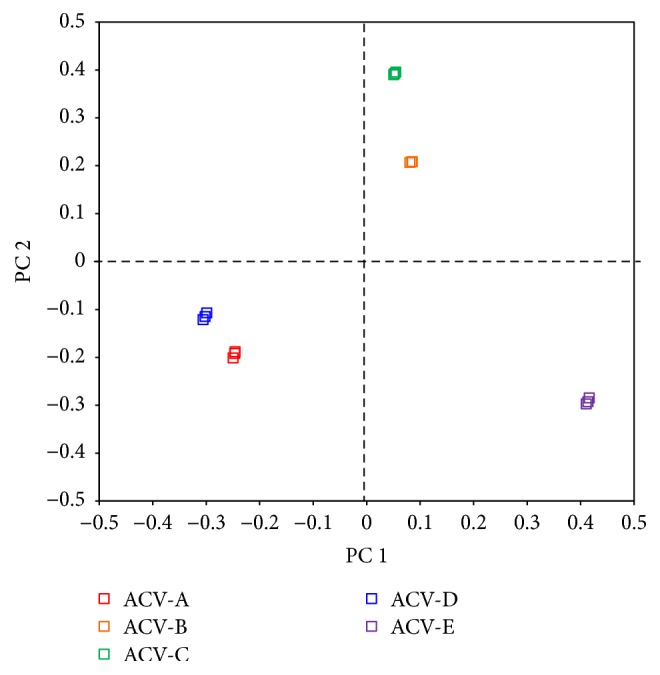
Principal component analysis of ACV ointments.

To evaluate differences in the principal component of each formulation, a principal component analysis was performed (Figure 7) [16]. In principal component 1 (PC1), ACV-A and ACV-D were similar, ACV-B and ACV-C were similar, and ACV-E differed from other ointments. In principal component 2 (PC2), ACV-A and ACV-D were similar, ACV-B and ACV-C were similar, and ACV-E differed from other ointments. Moisture content analysis and NIR absorption spectroscopy suggested that PC1 reflected differences in the water content of each formulation. PC2 differed between the five formulations. PC2 was considered to reflect differences in the molecular weight and the content of oleaginous bases and macrogol in ACV ointments. These results indicate that ointment spreadability is due to the moisture content of each formulation and the molecular weight and content of macrogol contained in each ointment. In addition, the viscosity and viscoelasticity of ointments were due to the molecular weight and content of macrogol contained in each ointment. Generally, macrogol is known to have different properties depending on the molecular weight of macrogol. For example, macrogol 400 is liquid and macrogol 4000 is solid at normal temperature, and the viscosity of macrogol 400 is lower compared with the viscosity of macrogol 4000. Therefore, the results of spreadability, viscosity, and viscoelasticity are considered due to the difference of the molecular weight of macrogol and each macrogol content of each ointment. Comparing the additives contained in each ointment, ACV-B and ACV-C contain macrogol 4000. However, these results indicated that ACV-B and ACV-C were soft and extensible preparations compared to other ointments. Therefore, the properties of ACV-B and ACV-C were considered due to the difference of each macrogol content. In addition, the difference of the formulation process of each ointment was also considered to contribute to the properties of the ointment preparation. Furthermore, the molecular weight of macrogol in ACV-D has not been reported. However, the moisture content, spreadability, viscosity, and viscoelasticity of ACV-D were similar to those of ACV-A; therefore, the molecular weight of macrogol contained in ACV-D was presumed to be 300 and 1500, which was the molecular weight of macrogol contained in ACV-A. The molecular weight of macrogol in ACV-E as an additive has not been reported. However, from the viscosity measurement and the second derivative of NIR absorption spectra, the molecular weight of macrogol with ACV-E was inferred to be lower than that with ACV-B and ACV-C. However, generally, the viscosity of high-molecular-weight macrogol is higher than the viscosity of low-molecular-weight macrogol. Therefore, the properties of ACV-E were considered due to the difference of each macrogol content and the production process of each ointment.

### 3.6. Skin Friction Measurement

**Figure 8:**
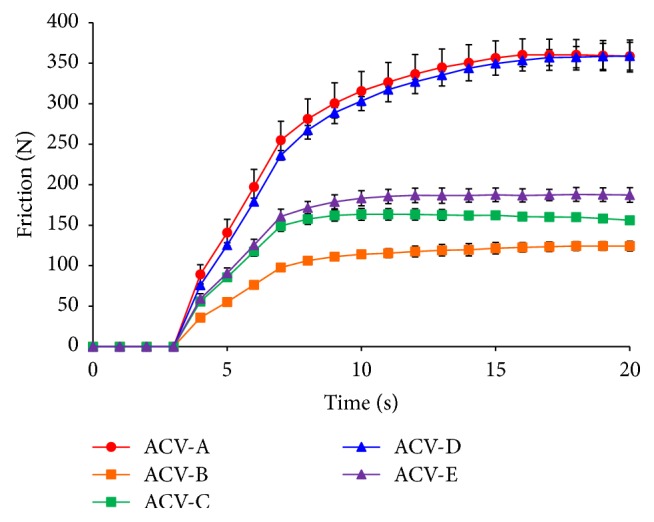
Skin friction of ACV ointments, respectively (n=5).

The Frictiometer was used to measure friction in order to evaluate the feeling of the creams when applied to the skin (Figure 8). This was also used to evaluate the feeling of cosmetics on the skin [17]. After 15 s, ACV-A had a friction measurement of 356.5 N, ACV-B had 121.6 N, ACV-C had 162.4 N, ACV-D had 349.7 N, and ACV-E had 187.5 N. The magnitude of the friction force followed the same pattern as the yield value calculated with a spread meter. The force of friction was inversely correlated with the spreadability and slipperiness of a semisolid preparation and was correlated with the stickiness of the preparation. Thus, ACV-B was inferred to be not sticky and to spread easily when applied to human skin compared with the other ointments.

## 4. Conclusion

A uniformity of content test revealed that ACV-A, ACV-B, ACV-C, ACV-D, and ACV-E possess equivalent ACV contents. Evaluation of the properties of each ointment revealed the physicochemical properties of ACV-A, ACV-B, ACV-C, ACV-D, and ACV-E. A skin friction test revealed differences in the properties of ACV-A, ACV-B, ACV-C, ACV-D, and ACV-E. This study investigated the properties of different ACV formulations in a model scenario involving a brand-name ointment, a generic ointment, and a switch OTC ointment. In the future, pharmacists will need to investigate the properties of OTC formulations. This will help maintain the health of the community and encourage appropriate self-medication.

## Figures and Tables

**Table 1:** Additive of ACV ointment each formulation.

Brand name	Lot number	Serial number	Additive
Zovirax® Ointment 5%	3E7F	ACV-A	Macrogol 300, macrogol 1500
Acyclovir Ointment 5% “TEVA”	CAO11	ACV-B	Macrogol 300, macrogol 400, macrogol 4000
Acyclovir Ointment 5% “TOWA”	A125	ACV-C	Macrogol 400, macrogol 4000, pH adjuster
Activir®	UW3M	ACV-D	Macrogol
Hifuru AC	Y503	ACV-E	Macrogol

**Table 2:** Content uniformity of ACV ointments.

Formulation	Uniformity (mean ± SD)
ACV-A	102.1 ± 2.0
ACV-B	100.8 ± 1.8
ACV-C	100.0 ± 1.4
ACV-D	103.1 ± 0.3
ACV-E	101.8 ± 0.8

Nonsignificant (n=3).

**Table 3:** Spreadability of ACV ointments at 25°C.

Formulation	Spreadability (mean ± SD)
ACV-A	22.9 ± 0.1
ACV-B	29.8 ± 0.2
ACV-C	26.5 ± 0.2
ACV-D	23.1 ± 0.3
ACV-E	23.8 ± 0.3

Value of the spreadability after 180 seconds (n=3).

## Data Availability

The data described in this article are not quoted from other studies and are new data obtained by this research. No data were used to support this study.
